# Transcriptome analysis of mesenteric arterioles changes and its mechanisms in cirrhotic rats with portal hypertension

**DOI:** 10.1186/s12864-023-09125-7

**Published:** 2023-01-14

**Authors:** Guangbo Wu, Min Chen, Qiang Fan, Hongjie Li, Zhifeng Zhao, Chihao Zhang, Meng Luo

**Affiliations:** Department of General Surgery, Shanghai Ninth People’s Hospital, Shanghai Jiao Tong University School of Medicine, Shanghai, 200011 China

**Keywords:** Liver cirrhosis, Portal hypertension, Splanchnic hyperdynamic circulation, Vascular changes, Mesenteric arterioles

## Abstract

Portal hypertension (PHT) is a major cause of liver cirrhosis. The formation of portosystemic collateral vessels and splanchnic vasodilation contribute to the development of hyperdynamic circulation, which in turn aggravates PHT and increases the risk of complications. To investigate the changes in mesenteric arterioles in PHT, cirrhotic rat models were established by ligating the common bile ducts. After 4 weeks, the cirrhotic rats suffered from severe PHT and splanchnic hyperdynamic circulation, characterized by increased portal pressure (PP), cardiac output (CO), cardiac index (CI), and superior mesenteric artery (SMA) flow. Mesenteric arterioles in cirrhotic rats displayed remarkable vasodilation, vascular remodeling, and hypocontractility. RNA sequencing was performed based on these findings. A total of 1,637 differentially expressed genes (DEGs) were detected, with 889 up-regulated and 748 down-regulated genes. Signaling pathways related to vascular changes were enriched, including the vascular endothelial growth factor (VEGF), phosphatidylinositol-3-kinase-AKT (PI3K-AKT), and nuclear factor kappa light chain enhancer of activated B cells (NF-κB) signaling pathway, among others. Moreover, the top ten hub genes were screened according to the degree nodes in the protein–protein interaction (PPI) network. Functional enrichment analyses indicated that the hub genes were involved in cell cycle regulation, mitosis, and cellular response to oxidative stress and nitric oxide (NO). In addition, promising candidate drugs for ameliorating PHT, such as resveratrol, were predicted based on hub genes. Taken together, our study highlighted remarkable changes in the mesenteric arterioles of cirrhotic rats with PHT. Transcriptome analyses revealed the potential molecular mechanisms of vascular changes in splanchnic hyperdynamic circulation.

**Supplementary Information:**

The online version contains supplementary material available at 10.1186/s12864-023-09125-7.

## Introduction

Portal hypertension (PHT) is a detrimental syndrome with persistently increased portal pressure (PP) caused by portal vein obstruction such as liver cirrhosis, portal vein thrombosis, and schistosomiasis [1, 2]. In liver cirrhosis, increased intrahepatic vascular resistance leads to PHT accompanied by the formation of portosystemic collateral vessels, ascites, and hyperdynamic circulatory syndrome [3]. In turn, hyperdynamic circulatory syndrome deteriorates PHT and induces related clinical complications. Esophageal variceal bleeding (EVB) is one fatal complication [4]. In advanced PHT, liver transplantation is the only effective treatment option.

In liver cirrhosis, the hyperdynamic circulatory syndrome of PHT is associated with splanchnic vascular changes. Excessive angiogenic factors are generated by extrahepatic vascular beds. For example, vascular endothelial growth factor (VEGF) leads to the formation of collateral vessels through the opening of existing vessels or angiogenesis [5]. Previous studies have demonstrated that portosystemic collaterals and angiogenesis are reduced by VEGF inhibition [6, 7]. On the other hand, vasodilation and arterial hypocontractility have occurred in PHT models. Nitric oxide (NO) is a vital factor in splanchnic vasodilation [2]. Although studies have reported that angiogenic and vasodilator factors play crucial roles in PHT, the mechanism of splanchnic hyperdynamic circulation has not been fully elucidated.

Previous studies have mostly focused on the effects of vasoactive substances on liver cirrhosis with PHT [8]. However, it is worth noting that impairment of neurons and vascular smooth muscle cells (VSMCs) is responsible for extrahepatic vascular changes. Hartl et al. found that neuropeptide Y improved vasoconstrictive activity by sensitizing VSMCs to norepinephrine (NE) and improving hyperdynamic circulation in cirrhotic rats [9]. Therefore, it is essential to investigate the specific mechanisms underlying splanchnic vascular dysfunction and remodeling in PHT.

In this study, we revealed vascular changes in the mesenteric arterioles of cirrhotic rats. RNA sequencing (RNA-Seq) was performed to identify differentially expressed genes (DEGs) in the mesenteric arterioles. Gene set enrichment analysis (GSEA) and protein–protein interaction (PPI) network were used to screen for potential pathways and hub genes. Moreover, based on hub genes, functional enrichment analyses were performed, and candidate drugs were predicted to ameliorate PHT in cirrhosis. Herein, for the first time, we aimed to reveal the molecular mechanisms of mesenteric arteriolar changes in cirrhotic rats through transcriptome analysis, which may provide a better understanding of the splanchnic hyperdynamic circulation of PHT.

## Materials and methods

### Animal models

Sprague Dawley (SD) rats (male, weight, 200–250 g, aged 6–8 weeks) were obtained from the Experimental Animal Center of the School of Medicine, Shanghai Jiao Tong University (Shanghai, China). Rats were housed in a specific pathogen-free facility under controlled conditions (humidity of 40–60%, 22 ℃ with a 12-h light/dark cycle) with free access to food and water. All SD rats were subjected to sham-operated (sham) or common bile duct ligation (BDL), as described previously [10]. The protocols for the rats were approved by the Ethical Committee of Shanghai Ninth People’s Hospital, Shanghai Jiao Tong University School of Medicine.

### Hemodynamic measurements

In accordance with our previous study [11], a PE50 tube was inserted into the portal vein or right femoral artery of rats following anesthesia with isoflurane (2%). The tube was then connected to a pressure transducer, and the PP, heart rate (HR), and mean arterial pressure (MAP) values were obtained (ALC-MPA multi-channel biological information analysis system, Shanghai Alcott Biotech Co., Ltd., Shanghai, China).

Cardiac output (CO) was determined using echocardiography [12]. SD rats were anesthetized by 2% isoflurane in induction boxes and placed in the supine position. The hair on the rat chest was removed with depilatory cream and washed with water for image acquisition. Next, the SMA flow was measured using a high‐frequency ultrasonography system (Vevo 2100 system, Fujifilm Visual Sonics, Toronto, Canada) with a pulsed Doppler transducer (Supplementary Fig. 1). The measurement of SMA flow rate was shown in detail in Supplementary Materials and Methods.

The stroke volume (SV) of each rat heart was calculated by dividing CO by HR (mL/beats). The rat cardiac index (CI, mL/min/ 100 g) was CO per 100 g of body weight. Systemic vascular resistance (SVR, mmHg/mL/min/100 g) was obtained by dividing MAP by CI. SMA resistance (mmHg/mL/min/100 g) was obtained using the (MAP-PP)/SMA flow per 100 g.

### Enzyme-linked immunosorbent assay (ELISA) and histological examination

According to the instructions of an ELISA kit (KRC3011, BMS625, Thermo Fisher, USA; SEKR-0071, Solarbio, China), the serum levels of tumor necrosis factor-α (TNF-α), interleukin-6 (IL-6) and IL-8 in rats were detected, respectively.

The right hepatic lobe and mesenteric arterioles were fixed in 10% formalin buffer (pH 7.4) and embedded in paraffin blocks. The liver and mesenteric arterioles were stained with hematoxylin and eosin (H&E). Masson’s trichrome and Sirius red staining were performed on liver sections. All tissue sections were randomly examined by an experienced pathologist under an optical microscope.

### Vascular function studies

As described in our previous study, the measurements of arterial contractility [13], stress-induced myogenic contraction of mesenteric arterioles [11], and the color microsphere method of portosystemic shunting (PSS) analysis [14] are shown in detail in the Supplementary Materials and Methods.

### RNA Sequencing of mesenteric arterioles

The mesenteric arterioles were isolated from sham (*n* = 6) and BDL rats (*n* = 6), and RNA-Seq was performed by TIANGEN Biotech (Beijing, China). Total RNA was extracted using TRNzol (TIANGEN). The concentration, purity and integrity of RNA samples. were determined using a Nano Drop 2000 spectrophotometer (Thermo Scientific, Wilmington, DE, USA) and an Agilent 2100 Bioanalyzer (Agilent Technologies, Santa Clara, CA, USA). 1 μg of total RNA was used to establish a paired-end RNA-seq library for transcriptome sequencing on an Illumina NovaSeq6000 platform and 150 bp paired-end reads were generated. After the low-quality reads had been trimmed and reads containing adapter had been removed, clean data was obtained for subsequent analyses. Details on workflow for RNA Sequencing are displayed in the Supplementary Materials and Methods.

### Identification of differentially expressed genes (DEGs)

Principal component analysis (PCA) was performed to determine the extent of similarity or dissimilarity between experimental groups based on overall mRNA expression profiles for each sample. The edgeR software (http://www.r-project.org/) was used to identify differentially expressed genes (DEGs). Prior to differential gene expression analysis, for each sequenced library, the read counts were adjusted by *edgeR* program package through one scaling normalized factor. Differential expression analysis was performed using the *edgeR* R package (3.18.1). The *p* values were adjusted using the Benjamini & Hochberg method. ∣log2 fold change∣ > 1 and *p* < 0.05 were set as the threshold for significantly differential expression. Volcanic plots were generated using the *ggplot2* R package. A heatmap was drawn using the *pheatmap* R package.

### Gene set enrichment analysis (GSEA)

Gene set enrichment analysis (GSEA) was a statistical methodology used to evaluate whether a given gene set was significantly enriched in a list of gene markers ranked by their correlation with a phenotype of interest [15]. GSEA was performed using the following software: (http://www.broadinstitute.org/gsea). The default weighted enrichment method was used for GSEA enrichment analysis.

### Protein–protein interaction (PPI) network

To explore the protein interactions and determine the hub genes, a protein–protein interaction (PPI) network of differentially expressed genes was constructed [16]. Construction of the PPI network was based on the STRING database, which is known to predict protein–protein interactions (http://string-db.org/). A combined score of interaction of > 0.4 was considered statistically significant. In this study, the PPI network was analyzed and visualized using Cytoscape (version 3.6.1). Molecular complex detection (MCODE, version 1.4.2) was used to determine the most critical modules. The centrality of the mRNA node was calculated by CytoHubba, and the hub genes were identified by the “MCC” method.

### Functional enrichment analyses

Gene Ontology (GO) annotation [17] and Kyoto Encyclopedia of Genes and Genomes (KEGG) [18] pathway analyses were performed using the *clusterProfiler* R package. Biological process (BP), cellular component (CC), and molecular function (MF) were included in GO terms. KEGG pathway analysis was used to illustrate gene functions and related signaling pathways. A *p*-value of < 0.05 was set as the cut-off criterion for GO and KEGG analyses.

### Quantitative Real-Time PCR (qRT-PCR)

Total RNA was extracted from the mesenteric arterioles using TRIzol reagent (15,596–026, Invitrogen, Carlsbad, CA, USA). All operations were performed at 4 °C or on ice following the manufacturer’s instructions. The RNA was then transferred to cDNA using the PrimeScriptTM RT reagent kit (RR047A, TAKARA, Shiga, Japan). The SYBR Green PCR Mastermix Kit (11201ES03, YEASEN, Shanghai, China) was used for PCR amplification on an ABI 7900HT Real-Time PCR system. The expression of RNA was standardized to β-actin mRNA levels. All experiments were performed in triplicates. The mRNA expression was calculated using the 2^−ΔΔCt^ method. The primer sequences of qRT-PCR used in this study were shown in Supplementary Table 1.

### Evaluation of candidate drugs

The DSigDB database was used to predict potential drug molecules based on hub genes [19]. The DSigDB database was implemented using the Enrichr platform (https://maayanlab.cloud/enrichment/), which has been widely applied to display multiple visualization details of the aggregation functions of genes [20].

### Statistical analysis

GraphPad Prism (version 7.0) and R language (version 4.0.4) were used for the above analyses. In this study, date for continuous variables were represented as mean ± standard deviation. Student’s *t*-test was used to evaluate differences between the two groups. Statistical significance was set as *p* < 0.05.

## Results

### Liver fibrosis and inflammation in BDL rats

**Fig. 1:**
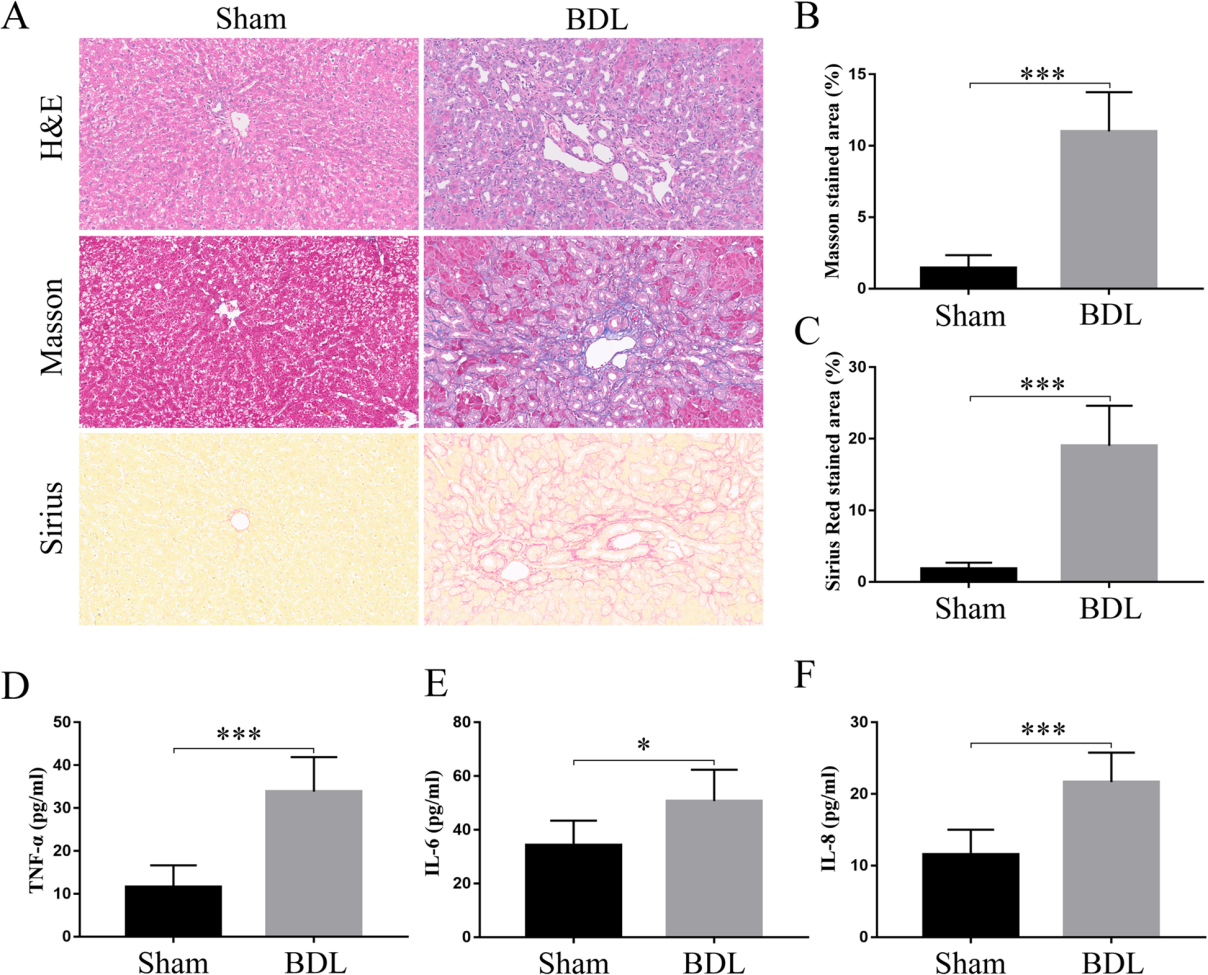
Liver fibrosis and inflammation were aggravated in BDL groups. **A** Representative figures of H&E staining, Masson's trichromatic staining and Sirius red staining. **B** and **C** Quantitative analysis of Masson and Sirius red staining in BDL rats revealed a marked increase in collagen deposition. **D**, **E** and **F** Serum levels of TNF-α, IL-6 and IL-8 were significantly increased in BDL rats. (Sham, *n* = 6, BDL, *n* = 6; *p < 0.05, ***p < 0.01)

Four weeks after operation, BDL surgery caused severe hepatocellular injury and liver fibrosis, and there were no pathological changes in sham-operated rats. H&E, Masson’s trichrome, and Sirius red staining were performed on liver sections (Fig. 1A). Subsequent quantitative analysis of BDL rats revealed a marked increase in collagen deposition (Fig. 1B and C). In addition, liver fibrosis was accompanied by the upregulation of inflammation. Serum levels of TNF-α, IL-6, and IL-8 were significantly increased in BDL rats (Fig. 1D-F). Thus, cirrhotic rat models with severe liver fibrosis and increased inflammation were successfully established.

### Body weight and hemodynamic parameters

In BDL rats, body weight was significantly decreased compared with that in sham-operated rats. The differences in hemodynamic parameters between the two groups are shown in Table 1. PP was markedly increased, and MAP was decreased in BDL rats (*p* < 0.01). PHT led to systemic and splanchnic arterial vasodilation and facilitated hyperdynamic circulation. CO, CI, and SMA flow were also markedly increased in BDL rats (CO, *p* = 0.06; CI, *p* = 0.01; SMA flow, *p* < 0.01). Meanwhile, SVR and SMA resistance in BDL rats were significantly decreased (*p* < 0.01).

**Table 1:** Body weight and hemodynamic parameters

Characteristics	Sham	BDL	Date	*p-*value
BW (g)	409.2 ± 7.0	366.7 ± 11.8	3.10	0.01
MAP (mmHg)	111.8 ± 2.8	89.8 ± 4.5	4.15	< 0.01
PP (mmHg)	6.7 ± 0.7	14.0 ± 1.1	5.84	< 0.01
HR (beats/min)	304.5 ± 18.4	334.5 ± 15.5	1.25	0.24
SV (mL/beats)	0.52 ± 0.03	0.61 ± 0.04	1.88	0.09
CO (mL/min)	158.6 ± 17.4	201.7 ± 9.7	2.16	0.06
CI (mL/min/100 g)	38.6 ± 3.8	55.4 ± 3.8	3.17	0.01
SVR (mmHg/mL/min/100 g)	3.0 ± 0.3	1.7 ± 0.1	4.17	< 0.01
SMA flow (mL/min/100 g)	2.6 ± 0.3	5.9 ± 0.3	7.63	< 0.01
SMA resistance				
(mmHg/mL/min/100 g)	42.0 ± 3.9	13.6 ± 1.0	7.06	< 0.01

### Vascular changes of mesenteric arterioles and PSS measurement in BDL rats

**Fig. 2:**
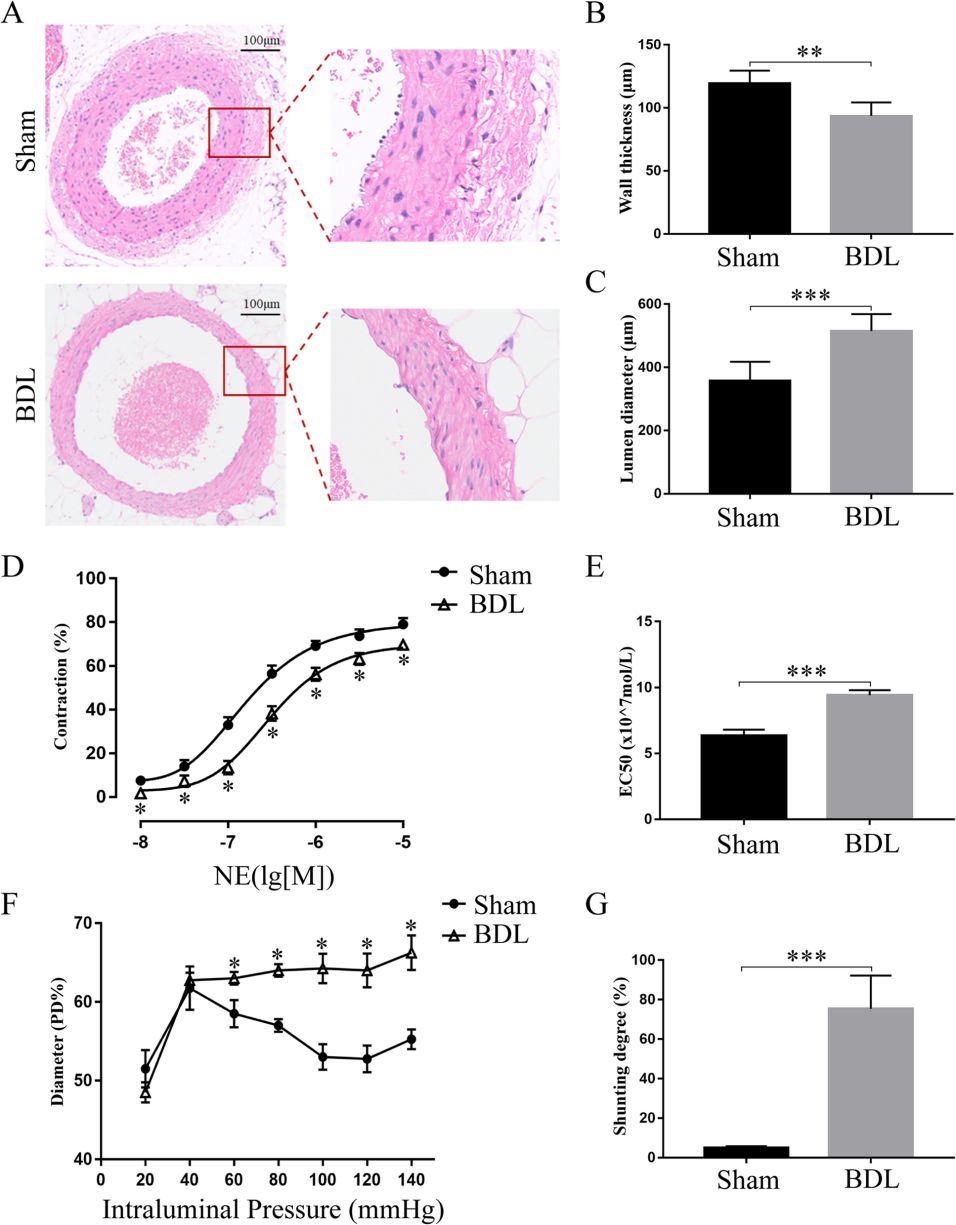
Vascular changes of mesenteric arterioles and portosystemic shunting (PSS) analysis. **A** Representative H&E staining figures of mesenteric arterioles. **B** Vascular wall of mesenteric arterioles became thinner, and **C** the lumen diameter was increased significantly in BDL rats. **D** and **E** The mesenteric arterioles of BDL rats showed remarkably hypo-responsiveness after NE exposure. **F** The stress-induced myogenic contraction was impaired in BDL rats. **G** Shunting degree: BDL rats had more severe PSS than sham operated rats. (Sham, *n* = 6, BDL, *n* = 6; *p < 0.05, ***p < 0.01)

The vascular structure and contractility of the mesenteric arterioles were investigated. As seen in Fig. 2A, there was significant vasodilation observed in BDL rats. The wall of the mesenteric arterioles became thinner, accompanied by slight structural disorders, and the lumen diameter increased significantly (Fig. 2B and C). Moreover, contractility of the mesenteric arterioles was assessed in both groups. Following NE exposure, there was remarkable hypo-responsiveness and hypocontractility of the mesenteric arterioles (NE EC_50_, 6.3 ± 0.3 vs 9.4 ± 0.2, in sham vs BDL rats, respectively, *P* < 0.01, Fig. 2D and E). Stress-induced myogenic contraction was impaired at 60–140 mmHg in BDL rats (Fig. 2F). In addition, the results of color microspheres showed that there was severe PSS in BDL rats compared with sham-operated rats (Fig. 2G).

### Identification of DEGs

**Fig. 3:**
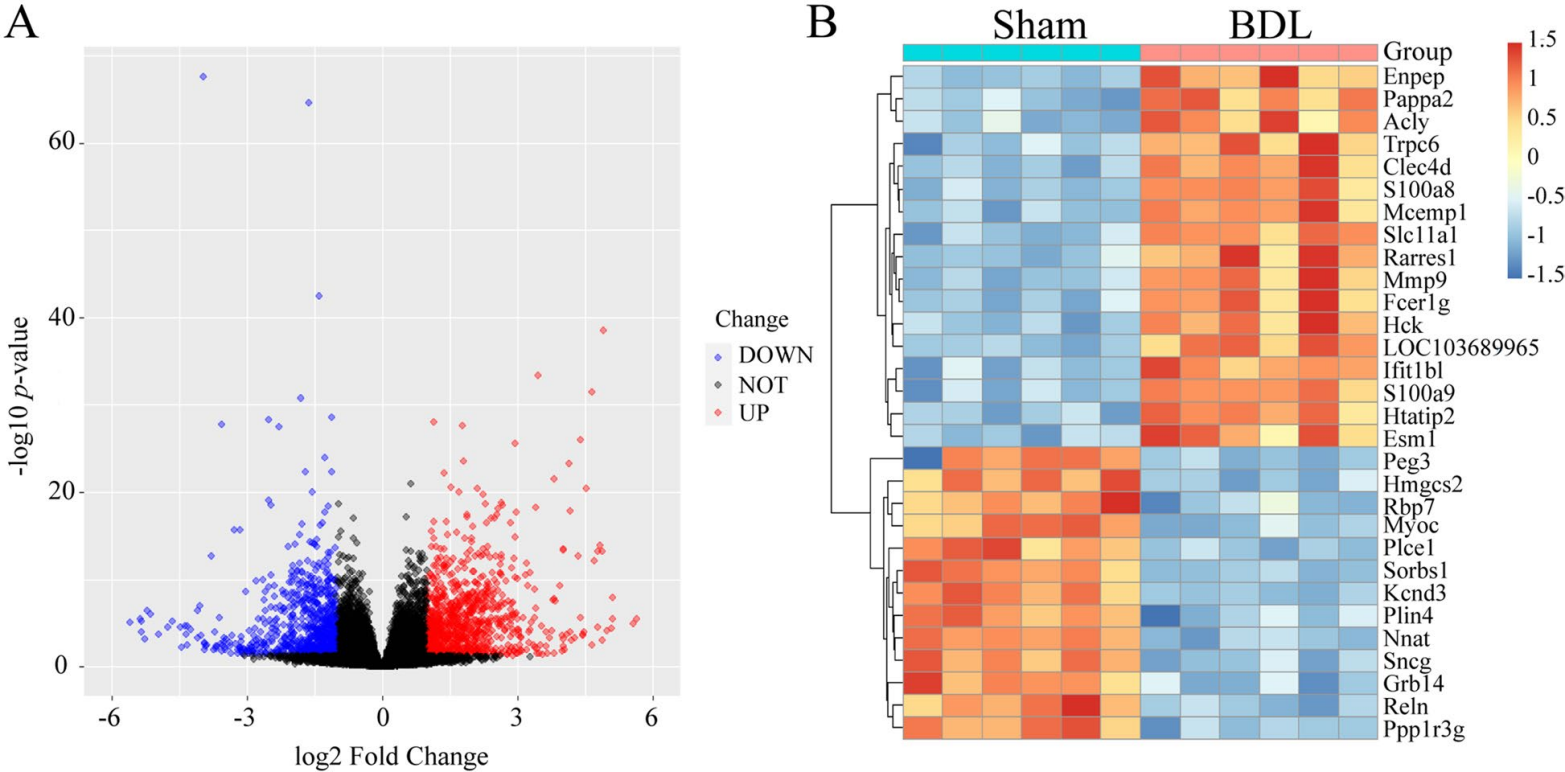
Identification of differentially expressed genes (DEGs) with the cut-off criteria:∣log2 fold change∣ > 1 and *p* < 0.05. **A** Volcano plots of DEGs with red for upregulated genes and blue for downregulated genes. **B** The heatmap showed the top 30 significant genes of DEGs

To reveal the molecular mechanisms of the vascular changes in BDL rats, mesenteric arterioles were collected for RNA-Seq. Vascular changes of BDL rats were linked to significant alterations in gene expression. PCA was used to analyze how individual samples relate to each other based on overall gene expression profiles. As shown in Supplementary Fig. 2, the red and green dots represented the sham and BDL groups respectively. More similar samples were clustered together, indicating that the intra-group difference was significantly smaller than the inter-group difference. A total of 1,637 DEGs were detected, of which 889 were upregulated and 748 were downregulated. As shown in the volcanic plots, DEGs are presented in the expression matrix, in which red and blue plots represent upregulated and downregulated genes, respectively (Fig. 3A). Subsequently, the top 30 significant DEGs are displayed in the heatmap (Fig. 3B).

### Enrichment analysis of GSEA

**Fig. 4:**
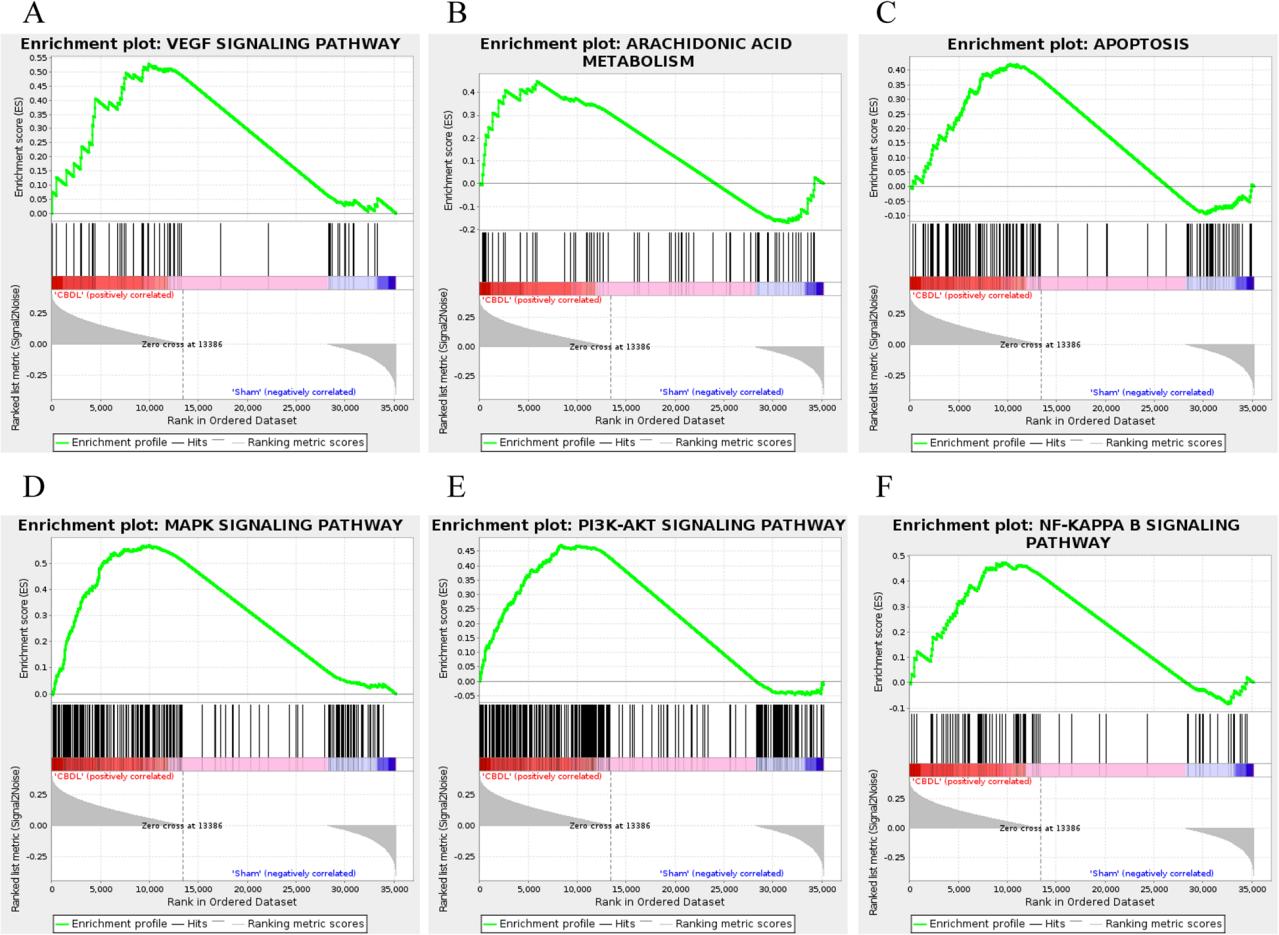
Gene Set Enrichment Analysis (GSEA) of DEGs. **A** VEGF signaling pathway. **B** Arachidonic acid metabolism. **C** Apoptosis. **D** MAPK signaling pathway. **E** PI3K-Akt signaling pathway. **F** NF-κB signaling pathway

GSEA was used to identify the potential functions and pathways of DEGs. GSEA results revealed that the vascular endothelial growth factor (VEGF) signaling pathway, arachidonic acid metabolism, apoptosis, mitogen-activated protein kinase (MAPK) signaling pathway, phosphatidylinositol-3-kinase-AKT (PI3K-AKT) signaling pathway, and nuclear factor kappa light chain enhancer of activated B cells (NF-κB) signaling pathway were all significantly enriched in BDL rats (Fig. 4A-F). They were responsible for alterations in vessels, including collateral angiogenesis, vascular remodeling, vasodilatation, and hypocontractility. Furthermore, in GSEA, there were other underlying signaling pathways involved in vascular changes, such as the Notch signaling pathway, Wnt signaling pathway, and renin secretion, which are shown in Supplementary Fig. 3.

### Construction of PPI network and correlation heatmap

**Fig. 5:**
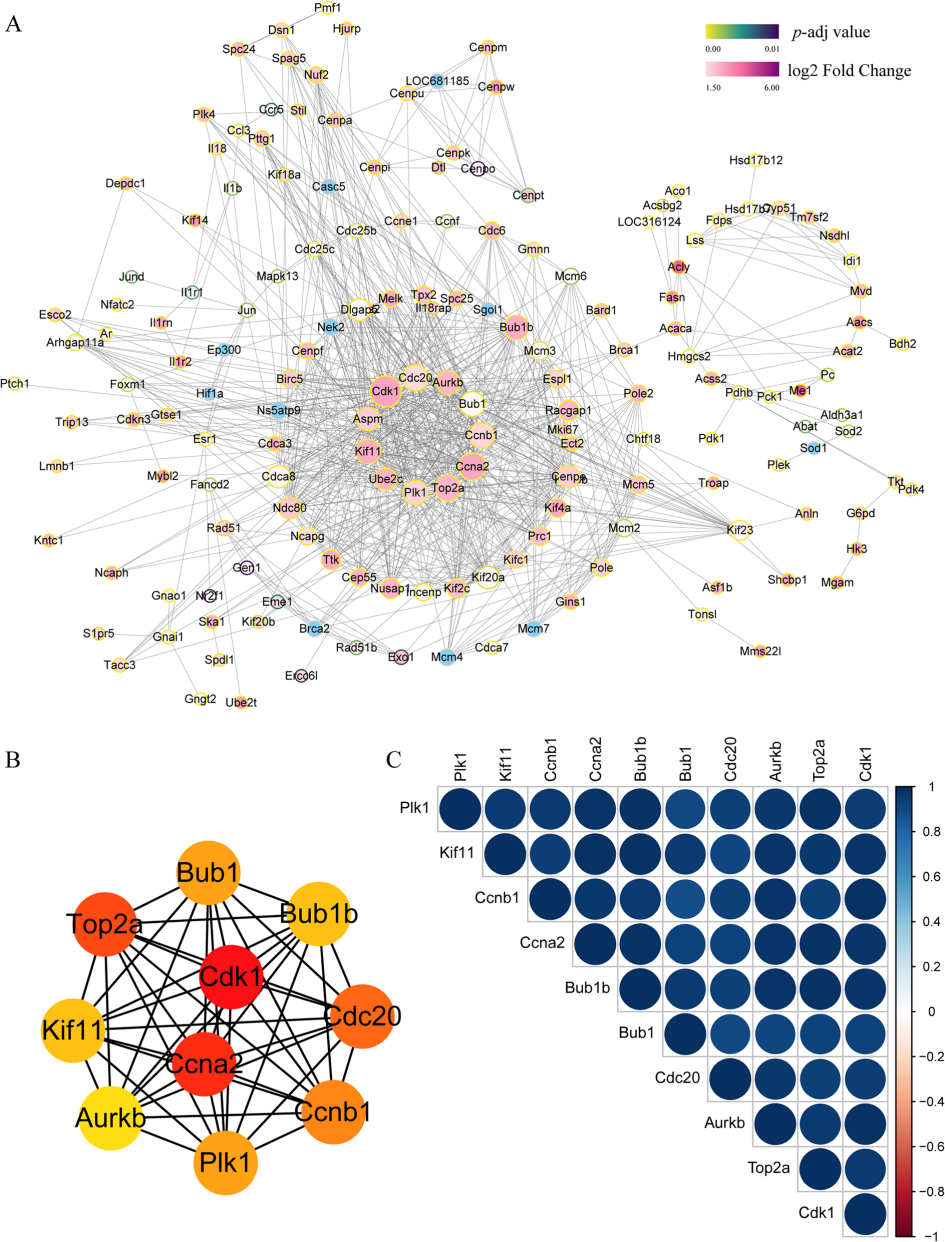
Construction of PPI network and Correlation heatmap. **A** The PPI network of DEGs was constructed with 170 interaction nodes. **B** The identification of top 10 hub genes determined by degree centrality. **C** The correlation of the 10 hub genes in the heatmap. Blue: positive correlation

To elucidate the specific molecular mechanism and identify hub genes, the DEGs were further filtered based on *p* values and fold change values. A total of 673 genes were used as input data and taken into PPI analysis. Then, the PPI network was constructed, and 170 interaction nodes were identified. As shown in Fig. 5A, the major gene clusters were identified based on GO analysis. In the most abundant gene cluster, the top ten hub genes were screened out according to the degree nodes, including Cdk1, Ccna2, Top2a, Cdc20, Ccnb1, Plk1, Bub1, Bub1b, Kif11, and Aurkb (Fig. 5B). The biological functions of the hub genes are listed in Supplementary Table 2. Subsequently, we performed a correlation analysis to determine the potential synergy or antagonism of each hub gene. In the correlation heatmap, there was a significant positive correlation among the ten hub genes, which is represented in blue (Fig. 5C).

### Expression of hub genes in qRT-PCR

**Fig. 6:**
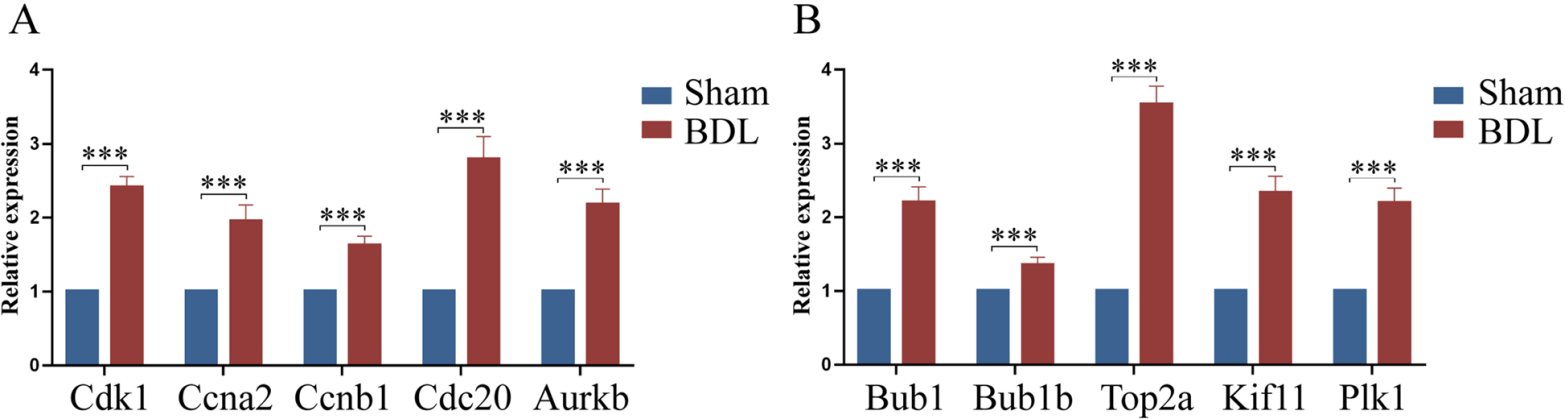
The expression levels of hub genes in mesenteric arterioles. **A** and **B** The mRNAs expression levels of 10 hub genes in qRT-PCR analysis. The expression of RNA was standardized to β-actin mRNA levels. (*p < 0.05, ***p < 0.01)

To validate the DEGs identified by RNA-seq analysis, the expression levels of the hub genes (Cdk1, Ccna2, Top2a, Cdc20, Ccnb1, Plk1, Bub1, Bub1b, Kif11, and Aurkb) were determined by qRT-PCR. As shown in Fig. 6A and B, there was significant up-regulation of the hub genes in BDL rats as compared with sham-operated rats. These findings were consistent with the transcriptome data. Thus, the results suggested that RNA-Seq reliably identified DEGs in the rat mesenteric arterioles.

### Functional enrichment analyses of hub genes

**Fig. 7:**
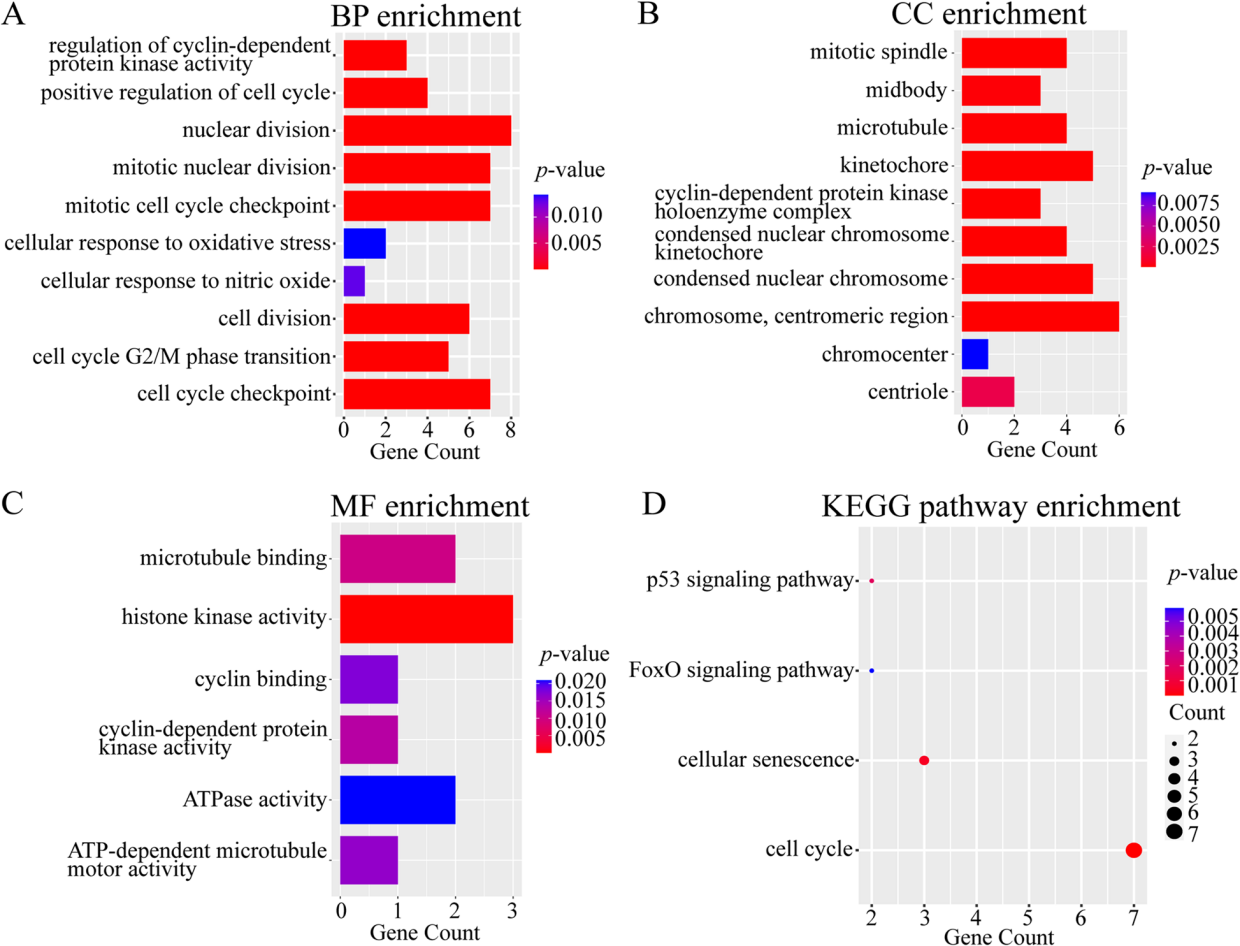
Functional enrichment analyses of hub genes. **A**,** B** and **C** The top enriched Gene ontology (GO) terms in biological process (BP), cellular component (CC) and molecular function (MF). The x-axis indicated gene counts, y-axis represented different GO terms, and the color of column represented *p* values. **D** The enriched pathways in KEGG analysis. Pathway date modified from KEGG pathway database (www.kegg.jp/kegg/kegg1.html). The x-axis indicated gene counts, y-axis represented different terms, and the color of dots indicated *p* values

GO and KEGG analyses were used to explore the underlying functions of hub genes. In GO BP analysis, the hub genes were enriched in cell cycle regulation and checkpoints, nuclear division, cellular response to oxidative stress, and cellular response to nitric oxide (NO) (Fig. 7A). The enrichment of CC terms included mitotic spindle, midbody, microtubule, condensed chromosome, and centriole (Fig. 7B). Additionally, the enrichment of MF terms included microtubule binding, histone kinase activity, cyclin binding, cyclin-dependent protein kinase activity, and ATPase activity (Fig. 7C). In addition, KEGG analysis was used to identify enriched pathways. According to the hub genes, there were four enriched pathways, including the p53 signaling pathway, FoxO signaling pathway, cellular senescence, and cell cycle (Fig. 7D). The number of genes involved in the cell cycle pathway was the highest in KEGG analysis.

### Identification of candidate drugs

**Fig. 8:**
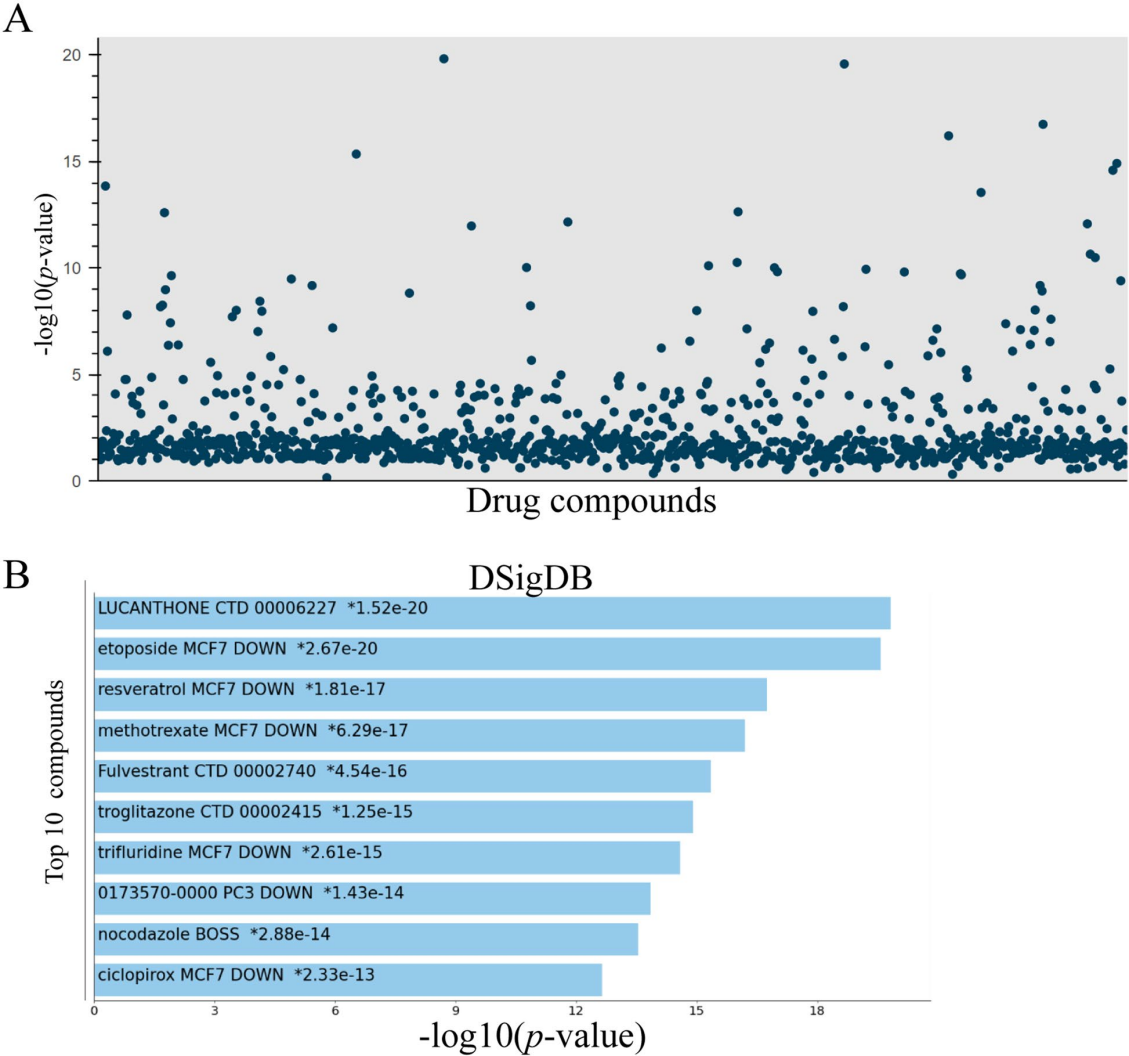
Identification of candidate drugs. **A** Drug compounds were predicted to ameliorate PHT. **B** The top 10 most significant candidate compounds were presented

Based on the ten hub genes, promising candidate drugs for ameliorating PHT were predicted using the DSigDB database. A large number of potential drug molecules were screened, among which the top ten most significant candidate drugs are presented in Fig. 8A and B. Candidate drug names, *p*-values, *q*-values, and overlap genes are displayed in Table 2. It is worth noting that resveratrol, a common antioxidant drug, included eight overlapping genes with high comprehensive scores.

**Table 2:** The top 10 candidate drug compounds

Term	*p-*value	q*-*value	Overlap genes
LUCANTHONE CTD 00,006,227	1.52e-20	1.39e-17	CCNA2, TOP2A, CDC20, CCNB1, PLK1, CDK1, BUB1B, KIF11, BUB1, AURKB
Etoposide MCF7 DOWN	2.67e-20	1.39e-17	CCNA2, CDC20, CCNB1, PLK1, BUB1B, KIF11, BUB1, AURKB
Resveratrol MCF7 DOWN	1.81e-17	6.32e-15	CCNA2, TOP2A, CDC20, CCNB1, PLK1, BUB1B, KIF11, BUB1
Methotrexate MCF7 DOWN	6.29e-17	1.64e-14	CCNA2, TOP2A, CDC20, CCNB1, PLK1, KIF11, BUB1
Fulvestrant CTD 00,002,740	4.54e-16	9.49e-14	CCNA2, TOP2A, CDC20, CCNB1, PLK1, CDK1, BUB1B, BUB1, AURKB
Troglitazone CTD 00,002,415	1.25e-15	2.17e-13	CCNA2, TOP2A, CDC20, CCNB1, PLK1, CDK1, BUB1B, KIF11, BUB1, AURKB
Trifluridine MCF7 DOWN	2.61e-15	3.89e-13	TOP2A, CDC20, CCNB1, PLK1, BUB1B, BUB1
0,173,570–0000 PC3 DOWN	1.43e-14	1.87e-12	CCNA2, CDC20, CCNB1, PLK1, KIF11, BUB1
Nocodazole BOSS	2.88e-14	3.35e-12	CCNB1, CDK1, BUB1B, KIF11, BUB1, AURKB
Ciclopirox MCF7 DOWN	2.33e-13	2.43e-11	TOP2A, CDC20, CCNB1, PLK1, BUB1B, BUB1

## Discussion

In liver cirrhosis, splanchnic hyperdynamic circulation can increase portal blood flow, leading to portal pressure aggravation. Although splanchnic vascular changes have been concerned over recent decades, the underlying molecular mechanism remains to be clarified. In this study, we revealed the vascular changes in the mesenteric arterioles of BDL rats, including vasodilation, vascular remodeling, and hypocontractility. The DEGs and enrichment signaling pathways associated with angiogenesis, vasodilation, inflammation, and cell cycle were identified by RNA-Seq. Moreover, our results showed that ten hub genes contributed to the splanchnic vascular changes caused by PHT, which might provide new targets for alleviating hyperdynamic circulation. It is interesting to note that drug compounds such as LUCANTHONE, resveratrol and troglitazone were predicted to be promising candidates for ameliorating PHT. As far as we know, this is the first study to report transcriptome analysis of mesenteric arterioles in BDL rats with PHT. Understanding the potential mechanism of structural and functional changes of mesenteric arterioles in cirrhotic rats contributes to establish strategies for improving PHT treatment.

According to the results of GSEA analysis, VEGF signaling pathway and Arachidonic acid (AA) metabolism were enriched in mesenteric arterioles of BDL rats. Our findings revealed that angiogenic factors, vasodilators, and inflammation were crucial promoters in the development of PHT in cirrhosis. Previous evidence has shown significant angiogenesis and vasodilation in mesenteric vascular beds, accompanied by increased VEGF and endothelial nitric oxide synthase (eNOS) levels [21]. Our previous study confirmed that high levels of NO were detected in vitro-perfused mesenteric arterioles of PHT rats [22]. Moreover, there are other excessive vasodilators such as carbon monoxide (CO), prostacyclin (PGI2), and epoxyeicosatrienoic acids (EETs) [23, 24]. Notably, it is consistent with our findings that AA metabolism plays a critical role in vascular changes in cirrhotic rats. A clinical study reported that plasma levels of EETs in cirrhotic patients were elevated, leading to increased skin blood flow [25]. Our recent study also found that hypocontractility of mesenteric arterioles was improved by reducing EETs in cirrhotic rats [13]. In contrast, AA metabolism plays a key role in mediating inflammatory responses. As demonstrated in our previous study, mesenteric vascular remodeling and angiogenesis were suppressed by the inhibition of cyclooxygenase-2 (COX-2) and soluble epoxide hydrolase (sEH) [26].

The complex pathological process of extrahepatic vascular changes in cirrhosis involves multiple signaling pathways. The results of this study showed that PI3K-Akt, MAPK, NF-κB and Interleukin-17 (IL-17) signaling pathways were enriched by GSEA analysis. Hepatopulmonary syndrome (HPS) is a complication of cirrhosis, characterized by vascular changes in intrapulmonary shunts and hypoxia. Rapamycin improved the inflammatory response and angiogenesis in HPS by inhibiting the VEGF and NF-κB signaling pathways [27]. Previous studies have shown that tumor necrosis factor-α (TNF-α) plays a key role in the development of HPS. Inhibition of TNF-α-mediated NO synthesis ameliorates HPS and hyperdynamic circulation via the PI3K-Akt pathway in cirrhotic rats [28].

Unlike previous studies, we do not limit our study to vasoactive substances, although they are essential for extrahepatic vascular changes. The subject of structural and functional changes in the mesenteric arterioles has aroused the interest of our research group. Our results confirmed that vascular remodeling and hypocontractility of mesenteric arterioles occurred in BDL rats. Together with the observation that inflammatory responses related pathways including MAPK, NF-κB and IL-17 signaling pathway were enriched in mesenteric vascular beds. Interestingly, we observed that similar pathological processes also occurred in intracranial aneurysms (IA). Owing to endothelial injury, the inflammatory response resulted in remarkable remodeling of the cerebral arteries [29]. Contractile VSMCs adjusted their phenotypes to migratory-proliferative and synthetic, which were characterized by the upregulation of inflammatory factors and cyclins as well as the downregulation of contractile proteins [30–32]. Li et al. reported that phenotype switching of VSMCs resulted in the formation of IA by destroying vascular structures and remodeling the extracellular matrix [33].

Additionally, in the present study, our results showed that apoptosis, cellular senescence and p53 signaling pathways were identified in the mesenteric arterioles. Furthermore, in the PPI analysis, the ten hub genes (Cdk1, Ccna2, Top2a, Cdc20, Ccnb1, Plk1, Bub1, Bub1b, Kif11, and Aurkb) were screened out, which were related to the regulation of cell cycle and mitosis. Given vascular remodeling and hypocontractility in BDL rats, it was tempting to speculate that phenotypic switching of VSMCs might occur in the mesenteric arterioles of cirrhotic rats. VSMCs are not terminally differentiated cells and exhibit plasticity in response to stimuli [32]. In this study, we proposed that the dedifferentiation of VSMCs lost its contractibility, which might lead to arterial vasodilation and hyperdynamic circulation in PHT. Nevertheless, the plasticity of VSMCs is diverse and heterogeneous. Multiple stimuli are involved in the regulation of phenotypic transition. There are complex mechanisms for phenotypic switching of VSMCs in different vascular diseases [34]. More experiments and direct evidence are needed to support the hypothesis that phenotypic switching of VSMCs in cirrhotic rats is responsible for splanchnic vascular changes.

There are increased levels of inflammatory factors and reactive oxygen species (ROS) in liver cirrhosis [35, 36]. In addition to the enrichment of inflammatory signaling pathways, the BP enrichment analysis in our study also identified hub genes involved in cellular response to oxidative stress. Vascular endothelial cell dysfunction could be induced by inflammation and ROS, which acted as initiators of phenotypic switching of VSMCs and vascular remodeling. As indicated in our previous studies, contractability of mesenteric arterioles to NE was improved after reducing hydrogen peroxide (H_2_O_2_) levels in cirrhotic rats [13]. Moreover, in our study, we found that Polo-like kinase 1 (Plk1), one of the hub genes, was not only an essential mitotic kinase in mitotic progression, but also a promoter that induced oxidative stress. As demonstrated by previous studies, the oxidative stress reaction was enhanced by Plk1, leading to endothelial cell dysfunction and the destruction of vascular homeostasis [37, 38].

Although the pharmacological treatment of cirrhosis has been studied for many years, there is still a lack of promising drugs to alleviate PHT and its complications. In the current study, the hub genes were used to predict potential drug molecules based on the DSigDB database. Notably, we found that resveratrol was screened as a potential drug to relieve PHT. Resveratrol alleviates oxidative stress and improves vascular endothelial function [39]. PHT in cirrhotic rats was improved by reducing O_2_^−^ levels and scavenging free radicals after resveratrol administration [35, 40]. Remarkably, resveratrol has exerted vascular protective effects and moderately lowered systolic blood pressure in patients in previous clinical studies [41–43]. However, the specific molecular mechanism of resveratrol in improving splanchnic hyperdynamic circulation of PHT requires further investigation.

There are several limitations in our study. First, only one cirrhotic rat model was used for RNA-Seq. Other liver cirrhotic models at different stages may also need to be explored. Second, the crucial molecular mechanisms of the VSMCs phenotype transition in BDL rats have not yet been elucidated. Finally, in the future, it is necessary to verify the efficacy of candidate drugs, especially resveratrol, in alleviating hyperdynamic circulation of liver cirrhosis.

## Conclusion

In summary, this study highlights the key roles of structural and functional changes in the mesenteric arterioles in the development of PHT. We comprehensively revealed differentially expressed mRNAs and important pathways between the BDL and sham groups. Ten hub genes (Cdk1, Ccna2, Top2a, Cdc20, Ccnb1, Plk1, Bub1, Bub1b, Kif11, and Aurkb) were screened and found to be involved in the cell cycle, mitosis, and cellular senescence, which might provide new targets for improving hyperdynamic circulation. Further, candidate drugs such as LUCANTHONE, resveratrol and troglitazone were predicted to alleviate PHT and its complications.

## Supplementary Information

**Additional file 1.** 

## Data Availability

The raw sequence data are available in the Sequence Read Archive (SRA) of the National Center for Biotechnology Information (NCBI) under the BioProject accession number of PRJNA859251: https://www.ncbi.nlm.nih.gov/bioproject/PRJNA859251.
