# Supplementary material for: Transcriptome analysis of mesenteric arterioles changes and its mechanisms in cirrhotic rats with portal hypertension
Source: BMC Genomics. 2023 Jan 14;24:20. doi: 10.1186/s12864-023-09125-7 (PMC9840839; doi:10.1186/s12864-023-09125-7)
Supplement: Supplementary file 1 — Additional file 1. [file 12864_2023_9125_MOESM1_ESM.docx]

# Supplementary Information

# Materials and Methods

SMA Flow Assessments

The ultrasonography system (Vevo 2100 system, Fujifilm Visual Sonics, Toronto, Canada) was used to measure the SMA flow of rats. After isoflurane anesthesia in rats, splanchnic arteries were easily identified with the aid of color flow imaging. The SMA flow was measured at the proximal 1-2 cm segment of the SMA near to its origin from the aorta. Angle of insonation should be maintained as low as possible to minimize the errors in calculations of velocity^[1]^. The mean of the three luminal diameter (mm) and three blood flow velocity (mm/s) were obtained at each scan in an attempt to increase reproducibility^[2, 3]^. The SMA flow rate was then calculated using the formula: SMA flow rate = Luminal Cross Sectional Area * Blood Flow Velocity.

Vascular function studies

As described in our previous study, the measurements of arterial contractility^[4]^, stress-induced myogenic contraction of mesenteric arterioles^[5]^, and color microsphere method of portosystemic shunting (PSS) analysis^[6]^ were showed in detail.

Measurement of mesenteric arterioles contractility

The small intestine section was obtained and transferred into frozen oxygenated modified Krebs bicarbonate buffer, physiological salt solution (PSS). The PSS contained 118.5 mM NaCl, 1.2 mM KH_2_PO_4_, 2.5 mM CaCl_2_, 1.2 mM MgSO_4_, 4.7 mM KCl, 25 mM NaHCO_3_ and 11 mM dextrose. The rat mesenteric arteriole, with a diameter of about 140–200 μm, was isolated and cannulated with a vascular chamber. The prepared PSS (5% CO_2_ and 95% O_2_) was used to perfuse the mesenteric arterioles for 45 minutes. The pressure in the vessels was kept at 80 mmHg. After equilibration for 45 minutes, the spontaneous tone of vessels was appeared, which was a basal tone about 25% maximal diameter. Arterioles were challenged with 10^−6^M phenylephrine (PE). In our study, if an artery demonstrated leakage or failed to contract more than 20% to PE, it was abandoned. The integrity of the endothelium cells was detected by a rapid relaxation of acetylcholine (10^−6^M) in the artery precontracted with PE. Cumulative concentration and contraction response curves were drawn by sequential addition of norepinephrine (NE; 10^−8^M to 10^−5^M). The BA310 microscope camera system (Motic, Xiamen, Fujian, China) was used to detected the inner diameter of vessels.

Myogenic contraction of mesenteric arterioles

As described above, the mesenteric arteriole, with a diameter of about 140–200 μm, was isolated and cannulated with a vascular chamber. The pressure in the mesenteric arteriole gradually increased to 80 mmHg, then the vascular diameter developed to maximum value, which was known as the passive diameter (PD80). Next, vascular diameter was reduced to the equilibrium state for 60 minutes, which was known as the basal diameter (BD80). The pressure in the vessels was changed to 20 mmHg and maintained for 10 minutes. The vessels pressure was increased to 140 mmHg in increments of 20 mmHg. The vessels reached a constant diameter while maintaining it for 10 min in each step. The perfusion solution was replaced by Ca^2+^ free buffer solution with 1 mmol/L EGTA at the end of each experimental protocol. The PD value of vascular at each pressure increment was recorded after gradually increase of pressure. The passive and active vascular diameters at different pressures were standardized by the following formula: PD% = di/PD80 × 100%, where di was the inner diameter of vascular and PD80 was the diameter in Ca^2+^ free buffer solution at 80 mmHg^[7]^.

Color microsphere method of PSS.

In our experiment, there were three types of color microspheres (15 μm, Triton Technologies, California, USA) with peak absorption at 672 nm (blue), 530 nm (red) and 448 nm (yellow)^[8, 9]^. The yellow spheres were injected into the left ventricle and the red spheres were injected into the portal vein. In the process of microsphere administration, a reference blood sample was collected from the left femoral artery catheter at a constant speed of 0.65 mL/min with a suction syringe pump (ALC-IP 900, Alcott Biotech, Shanghai, China) for 1 minute. After the syringe pump was used for 10 seconds, 30,000 red and 300,000 yellow microspheres were suspended in 0.3 mL normal saline containing 0.05% Tween 80, respectively, and injected into the portal vein and left ventricle. After microsphere injection, the rats were sacrificed through bilateral thoracotomy, and we collected liver, stomach, lung, kidney, pancreas, colon, small intestine and mesenteric tissues from the rats. After the excess blood was removed and weighed, the tissue was boiled in a 5 M KOH solution containing 10% Tween 80 for complete digestion. The microspheres were collected after tissue digestion by centrifugation and washed in PBS. The color of microspheres was dissolved into 0.2 mL dimethylformamide and detected by spectrophotometry (BIO-RAD, California, USA) with respective peak absorptions. Blue microspheres were added to each sample as an internal control prior to the start of sample processing.

RNA Sequencing workflow

**RNA sample quantification and qualification**

Nanodrop 2000 spectrophotometer (Thermo Fisher Scientific) was used to determine the concentration and evaluate the purity of RNA samples. Agilent 2100 Bioanalyzer and 2100 RNA nano 6000 assay kit (Agilent Technologies) were used to evaluate the integrity of RNA samples.

**Transcriptome sequencing library preparation**

The RNA with poly-A in eukaryotic total RNA was enriched by TIANSeq mRNA Capture Kit (TIANGEN). Then, using the captured RNA as the starting sample, TIANSeq Fast RNA Library Kit (Illumina) was used to construct the transcriptome sequencing libraries. Briefly, the transcriptome sequencing library was constructed through RNA randomly fragmentation, cDNA strand 1 / strand 2 synthesis, end repair, A-tailing, ligation of sequencing adapters, size selection and library PCR enrichment.

**Library quantification and qualification**

Library concentration was first quantified using Qubit 2.0 fluorometer (Life Technologies), and then diluted to 1 ng/µl before checking insert size on an Agilent 2100 and quantifying to greater accuracy by quantitative PCR (Q-PCR) (library activity >2 nM).

**Clustering and sequencing**

The clustering of the index-coded samples was performed on a cBot Cluster Generation System using TruSeq PE Cluster Kit v3-cBot-HS (Illumina) according to the manufacturer’s instructions. After cluster generation, the library preparations were sequenced on an Illumina sequencing platform and 150 bp paired-end reads were generated. After the low-quality reads had been trimmed and reads containing adapter had been removed, clean data was obtained for subsequent analyses.

**Quality Control**

Raw data (raw reads) of FASTQ format were firstly processed through in-house perl scripts. In this step, clean data (clean reads) were obtained by removing the reads containing adaptors, reads with more than 10% poly-N and reads of low quality (the number of bases with sQ ≤ 5 accounts for more than 50% of the total read length) from the raw data. Trimmomatic (Version 0.36, http://www.usadellab.org/cms/index.php?page=trimmomatic) was used for quality trimming and filtering. At the same time, Q20, Q30 and GC content the clean data were calculated. All the downstream analyses were based on the clean data with high quality.

# Supplementary Tables

**Supplementary Table 1. Primer sequences of qRT-PCR**

| **Gene** | **Forward primer (5’------3’)** | **Reverse primer (5’------3’)** |
| --- | --- | --- |
| Cdk1 | CCAAGAAGCCGCTTTTCCAC | GCAGGGACTCTACTTCTGGC |
| Ccna2 | GGATGGTAGTTTTGAATCACCCC | GGATGGCCCGCATACTGTTA |
| Top2a | CCAAAGCCAAGAACAGTCGC | CACATGGAAATCCCGCTCCT |
| Cdc20 | CAAGGCTGTTGCATGGTGTC | ATGGAGCACACCTGGGAATG |
| Ccnb1 | GTGGAGCAGCATACTTTGGC | TGGTGTCCATTCACCGTTGT |
| Plk1 | TTCCGGAGGTCCTGATGGAT | TTCTGATGGGGCTTGAGCAG |
| Bub1 | ACAATACAGGCTATTCCAGGCA | CAGGCTTGGGTGCCATAGAT |
| Bub1b | AGTTCTCAATGCCCAGCGAA | TGAAACCGGTGATGATGGCA |
| Kif11 | CACGGGAGCAACTCCTTGAT | CGGGGTTAAGGCCTCTGTTT |
| Aurkb | TTGCAAGGATCCCAGAGCAG | TGGATTTCGATCTCTCGGCG |
| β-actin | CTCCATCCTGGCCTCGCTGT | GCTGTCACCTTCACCGTTCC |

**Supplementary Table 2. Biological functions of top 10 hub genes**

| Gene | Biological Functions |
| --- | --- |
| Cdk1 | A key role in the control of the eukaryotic cell cycle and required for entry into S-phase and mitosis |
| Ccna2 | Essential for the control of the cell cycle at the G1/S (start) and the G2/M (mitosis) transitions |
| Top2a | Regulation of topological states of DNA by transient breakage and subsequent rejoining of DNA strands |
| Cdc20 | Required for two microtubule-dependent processes, nuclear movement prior to anaphase and chromosome separation |
| Ccnb1 | Essential for the control of the cell cycle at the G2/M (mitosis) transition. |
| Plk1 | Serine/threonine-protein kinase that performs important functions throughout M phase of the cell cycle and regulates TP53 stability |
| Bub1 | Serine/threonine-protein kinase that performs crucial functions during mitosis, mediates cell death in response to chromosome missegregation |
| Bub1b | Essential component of the mitotic checkpoint |
| Kif11 | Motor protein required for establishing a bipolar spindle |
| Aurkb | Involved in regulating the cleavage of polar spindle microtubules and a key regulator for the onset of cytokinesis during mitosis |

# Supplementary Figures

**Supplementary Figure 1.** Doppler ultrasound measurements of superior mesenteric artery (SMA) flow. (**A**) The SMA diameter and blood flow rate in sham-operated rats. (**B**) The SMA diameter and blood flow rate were increased in BDL rats.


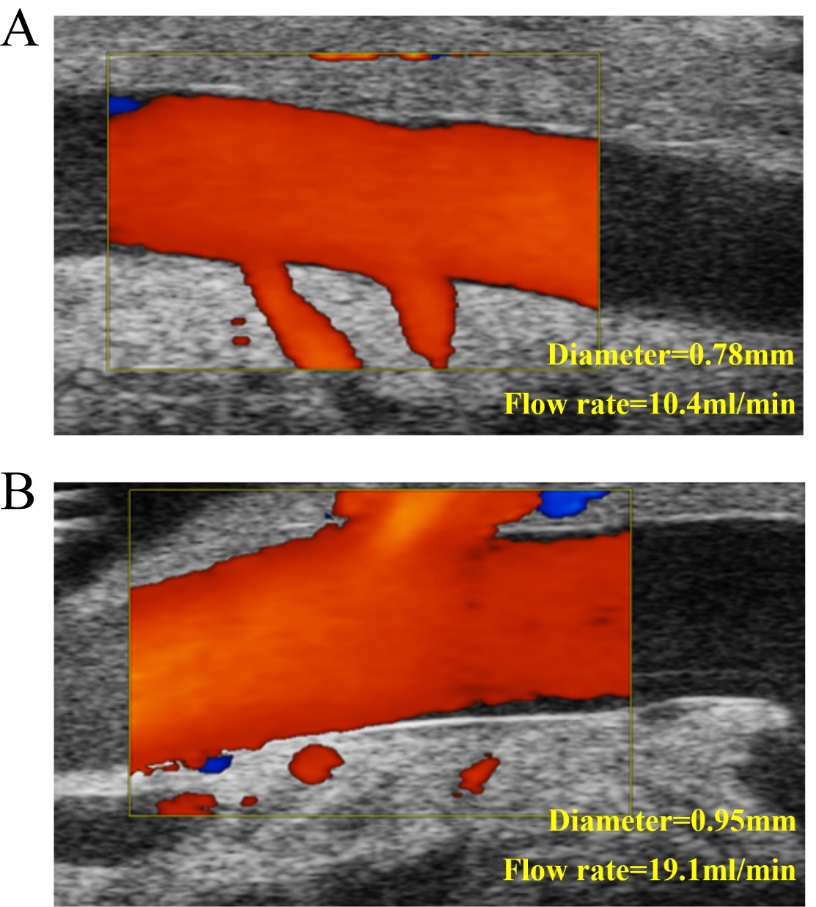


**Supplementary Figure 2.** A principal component analysis (PCA) of transcriptome data.


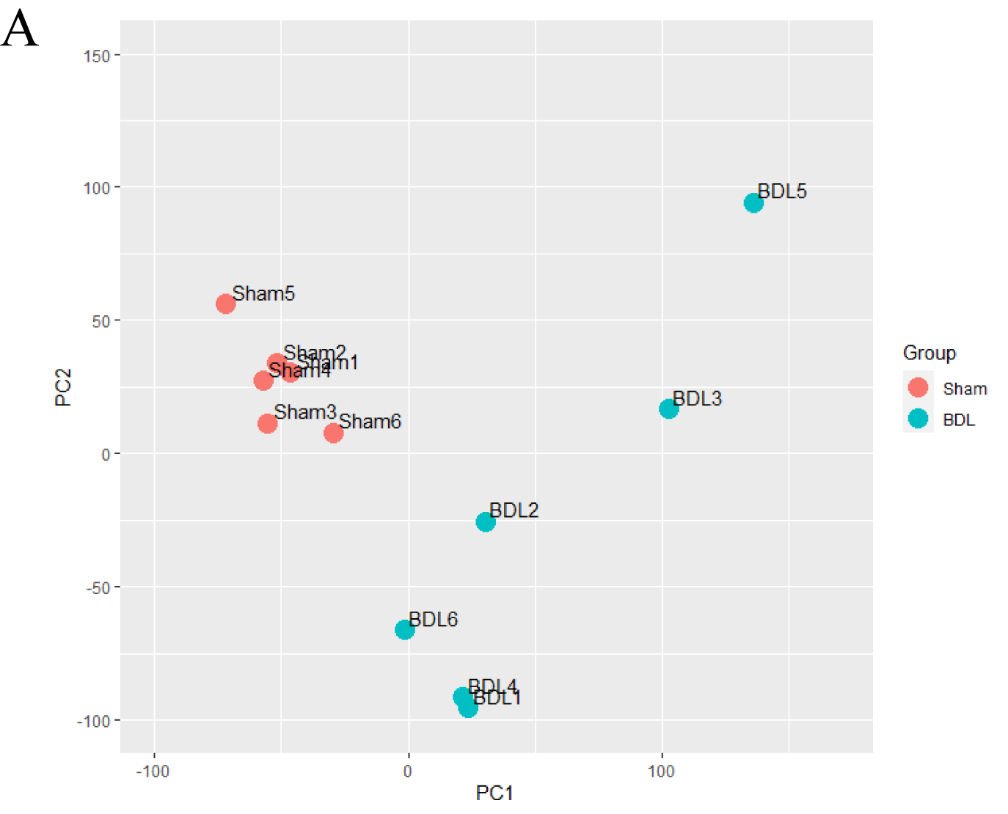


**Supplementary Figure 3.** Gene Set Enrichment Analysis (GSEA) of DEGs. (**A-I**) The potential signaling pathways associated with vascular changes were identified by GSEA.


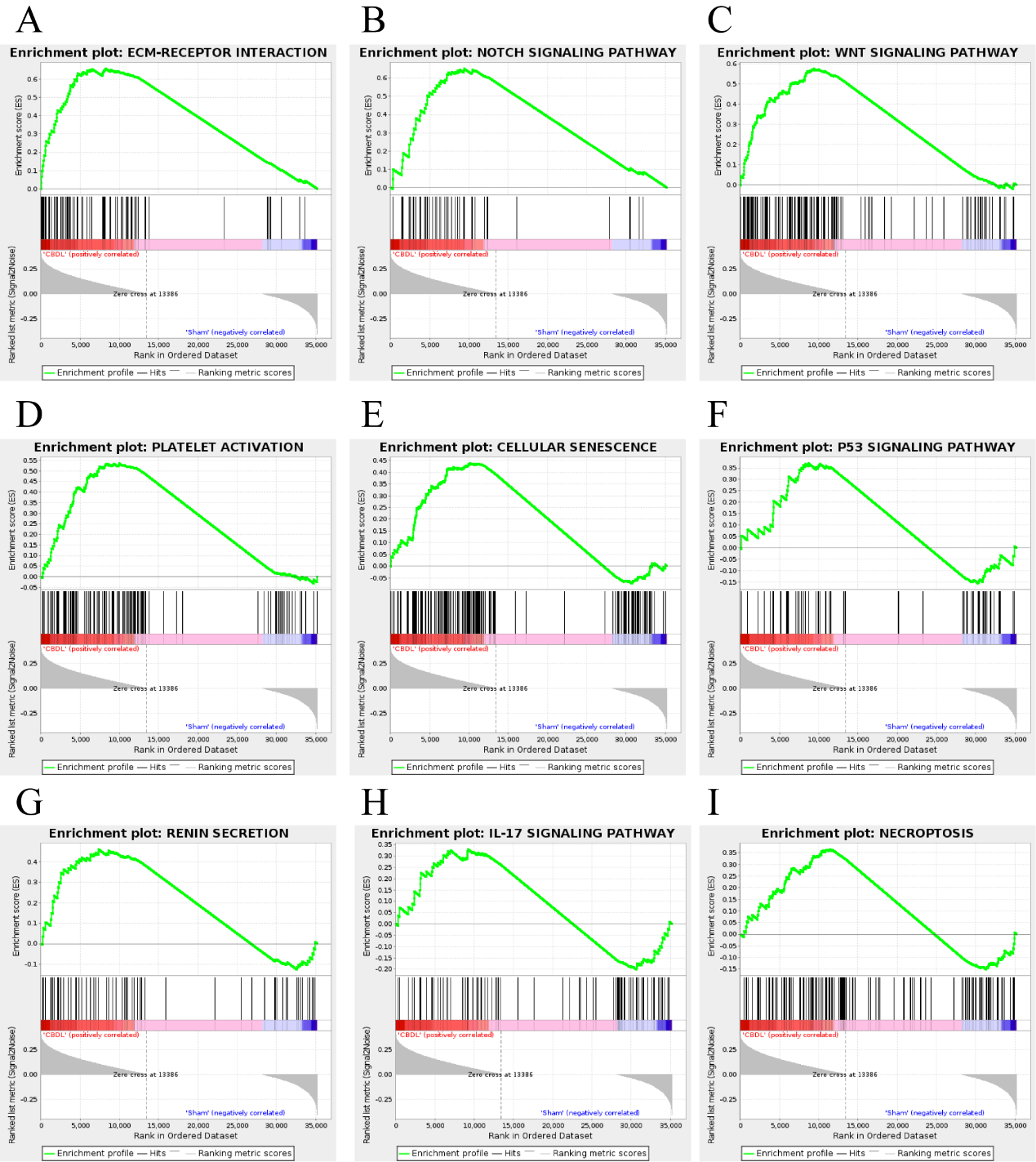


# Reference

[1] Gatt M, MacFie J, Anderson AD, et al. Changes in superior mesenteric artery blood flow after oral, enteral, and parenteral feeding in humans. Crit Care Med. 2009;37(1):171-6.

[2] Wu J, Agbor LN, Fang S, et al. Failure to vasodilate in response to salt loading blunts renal blood flow and causes salt-sensitive hypertension. Cardiovasc Res. 2021;117(1):308-19.

[3] Geelkerken RH, Lamers CB, Delahunt TA, et al. Duodenal meal stimulation leads to coeliac artery vasoconstriction and superior mesenteric artery vasodilatation: an intra-abdominal ultrasound study. Ultrasound Med Biol. 1998;24(9):1351-6.

[4] Wang X, Gu H, Li K, et al. DPP4 inhibitor reduces portal hypertension in cirrhotic rats by normalizing arterial hypocontractility. Life Sci. 2021;284:119895.

[5] Huang Y, Qin J, Sun D, et al. Inhibition of soluble epoxide hydrolase reduces portal pressure by protecting mesenteric artery myogenic responses in cirrhotic rats. Prostaglandins Other Lipid Mediat. 2017;131:17-24.

[6] Deng W, Duan M, Qian B, et al. NADPH oxidase 1/4 inhibition attenuates the portal hypertensive syndrome via modulation of mesenteric angiogenesis and arterial hyporeactivity in rats. Clin Res Hepatol Gastroenterol. 2019;43(3):255-65.

[7] Liu L, Liu H, Sun D, et al. Effects of H(2)S on myogenic responses in rat cerebral arterioles. Circ J. 2012;76(4):1012-9.

[8] Kowallik P, Schulz R, Guth BD, et al. Measurement of regional myocardial blood flow with multiple colored microspheres. Circulation. 1991;83(3):974-82.

[9] Hakkinen JP, Miller MW, Smith AH, et al. Measurement of organ blood flow with coloured microspheres in the rat. Cardiovasc Res. 1995;29(1):74-9.
